# Increased Gonadotropins and prolactin are linked to infertility in males

**DOI:** 10.6026/97320630016176

**Published:** 2020-02-29

**Authors:** Pravin Kumar Gangwar, Satya Narayan Sankhwar, Shriya Pant, Akhilesh Krishna, Bhupendra Pal Singh, Abbas Ali Mahdi, Rajender Singh

**Affiliations:** 1 Department of Urology, King George's Medical University, Lucknow, U.P.,India; 2 Department of Physiology, King George's Medical University, Lucknow, U.P.,India; 3 Department of Biochemistry, King George's Medical University, Lucknow, U.P.,India; 4 Division of Endocrinology, Central Drug Research Institute, Lucknow, U.P.,India

**Keywords:** Hormones, male infertility, spermatogenesis

## Abstract

Infertility has become a significant issue among married couples worldwide. The association of variations in reproductive hormones with infertility is evaluated at a tertiary care hospital in North India. A total of 220 infertile males having infertility longer than one year (cases) and 220 age-matched fertile males with confirmed paternity in past two to three years (controls) were enrolled for the study. Serum levels of LH, FSH, testosterone and PRL were measured by Roche e411 autoanalyzer using electrochemiluminescense immunoassay technique. Significant higher levels of serum hormone (mean±SD) were found in cases vs. controls; LH (9.02±7.81 vs. 5.22±1.45 mIU/ml), FSH (11.45±14.02 vs. 4.09±1.62 mIU/ml) and PRL (199.08±80.79 vs. 127.23±81.64 µIU/ml). However, the serum testosterone level was significantly low in cases associated with male infertility (4.62±2.03 vs. 6.82±2.79 ng/ml). LH, FSH and PRL levels were significantly increased in azoospermic, oligozoospermic and asthenozoospermic infertile males while FSH and PRL were significantly elevated in normozoospermic infertile group. Conversely, mean serum testosterone levels were significantly low in all infertile subgroups in comparison to fertile controls. PRL showed a significant prediction of Normozoospermia (AUC=0.836, Z=4.916, p<0.001) in ROC analysis. Data presented here is interesting, requiring further confirmation using larger samples of multiple cohorts.

## Background

Infertility is defined as the inability of a sexually active, non-contracepting couple to achieve a clinical pregnancy after 12 months or more of regular sexual intercourse [1]. Around 48.5 million couples were diagnosed with infertility worldwide [2]. It was also reported that approx. 30 million couples in India suffer from infertility [3]. Infertility affects both men and women but in developing countries, women are generally blamed. However, recent advances prove the equal contribution (40-50%) of male partners in overall infertility cases. Various factors like increasing age, diet, obesity, excessive smoking, and alcoholism affect the fertility status of men. Endocrinal factors also play an imperative role in the development and maintenance of reproductive processes. It is well established that normal male fertility is represented by the complete development of male germ cells and normal spermatogenesis, which occurs due to the balanced endocrine interplay of hypothalamic-pituitary-gonadal (HPG) axis. The Hypothalamus releases gonadotropin-releasing hormone (GnRH), stimulates the pituitary gland for the secretion of lutenizing hormone (LH) and follicle-stimulating hormone (FSH). FSH stimulates spermatogenesis directly by acting on seminiferous tubules in Sertoli cells whereas LH induces sperm production indirectly via testosterone synthesis in Leydig cells. Prolactin (PRL) hormone secreted by the pituitary, controls the production of LH and FSH via the regulation of GnRH through feedback mechanism on the hypothalamus. Ultimately, altered levels of serum hormones may create disturbances in spermatogenesis and cause infertility in males. Tests for serum hormones are to be required if there is any indication of abnormal semen analysis or any hormonal disorder. If the sperm-producing capacity of the testis is diminished, the pituitary starts producing more FSH to revive the normal testicular function. Therefore, a very high level of FSH indicates an abnormality in initial sperm production [4]. Moreover, increased FSH levels in azoospermia and severe-oligozoospermia are indicative of damaged seminiferous tubules [5]. Low semen quality is detected in patients with a higher level of LH, FSH and lower level of testosterone [6,7]. However, studies relating sperm motility with serum levels of PRL reported decreased or higher levels of the hormone in the individuals with asthenozoospermia but the excessive level of serum PRL was correlated with infertility, impotence and hypogonadism [8,9]. On the other hand few reports believed to have no role or the limited role of serum PRL in male infertility [10]. As several reports have been published on the role of different hormones in male infertility worldwide but very few of them have looked at variations in these serum hormone levels among different subtypes of male infertility in the North Indian population. Moreover, data regarding the association of different serum levels of PRL with male infertility is scanty and controversial. Therefore, the present study has been undertaken to comprehensively evaluate the variations in the serum levels of LH, FSH, testosterone and PRL and also determine their association with different types and subtypes of male infertility in north Indian patients.

## Materials and Methods:

### Study Design and Subjects:

The present case-control study was carried out between periods of 2014 to 2018. A total of 220 infertile males as cases and age-matched 220 fertile males as controls were enrolled from Department of Urology, after obtaining ethical clearance from the Institutional Ethics Committee, King George's Medical University (KGMU), Lucknow, U.P., India (Reference code number: 1163/R-Cell/12). Informed consent of each patient was obtained in response to a fully written and verbal explanation of the nature of the study. The inclusion criteria for infertile patients were based on infertility persisting longer than one year with the clinically fertile female partner. All the patients were clinically examined and information on demographical characteristics, family history, duration of the marriage, any health-related problem, parity and risk habits of all volunteers have been noted down on pre-designed history Proforma sheet. Male individuals exhibiting obstruction to sperm release, varicocele, any infection of accessory glands, human immunodeficiency virus positivity, diabetes, and any malignancy were excluded from this study. All the study participants belonged to north India with mean (± SD) age 31 ± 4.9 years for the case group and 29.9 ± 5.0 years for the control group.The semen analysis was performed after abstinence of 3-7 days. After examination of semen quality, the patients were categorized in different subtypes according to the WHO criteria [11]. The case group consisted of individuals with azoospermia (n=102), oligozoospermia (n=28), asthenozoospermia (n=76) and normozoospermia (n = 14). The controls were recruited following the criteria of confirmed paternity in the last two to three years.

### Estimation of serum hormones:

For the estimation of serum hormone levels, a blood sample from each individual of both groups has been obtained. The LH, FSH, testosterone and PRL levels were analyzed in blood serum and quantified by Roche e411 analyzer (Roche Diagnostics, USA) using electrochemiluminescense immunoassay technique. Normal reference ranges of LH, FSH, Testosterone and PRL for men were 1.7-8.6 mIU/ml, 1.5-12.5 mIU/ml, 2-8 ng/ml and 86-324 µIU/ml, respectively.

### ROC analysis Diagnostics:

The diagnostic of variables (LH, FSH, Testosterone, and PRL) to discriminate controls and male infertile cases (Normozoospermia, Oligozoospermia, Asthenozoospermia and Azoospermia) was done using ROC curve analysis.

### Statistical analysis:

Statistical analysis was performed by using the SPSS software tool (version 16; IBM corp., IL, USA). The categorical data were summarized as number and percentage and continuous data in mean ± standard deviation (SD). The chi-square test (χ^2^), unpaired t-test and ANOVA were used to assess the associations, and Pearson's correlation coefficient (r) showed the correlation between two parameters. The ROC curve (Receiver Operating Characteristics) analysis was done to access predictors for infertility. A two-tailed P <0.05 was considered as statistically significant.

## Results:

**Figure 1:**
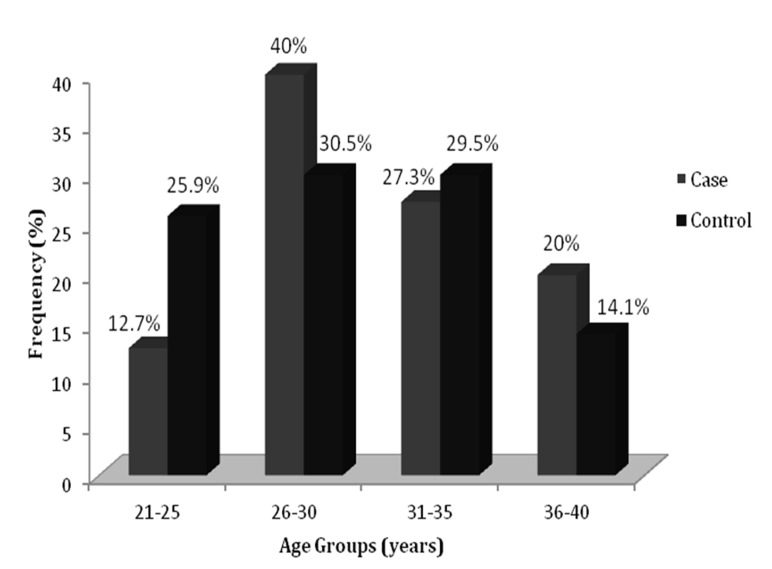
Frequency of infertile cases and controls according to age groups

**Figure 2:**
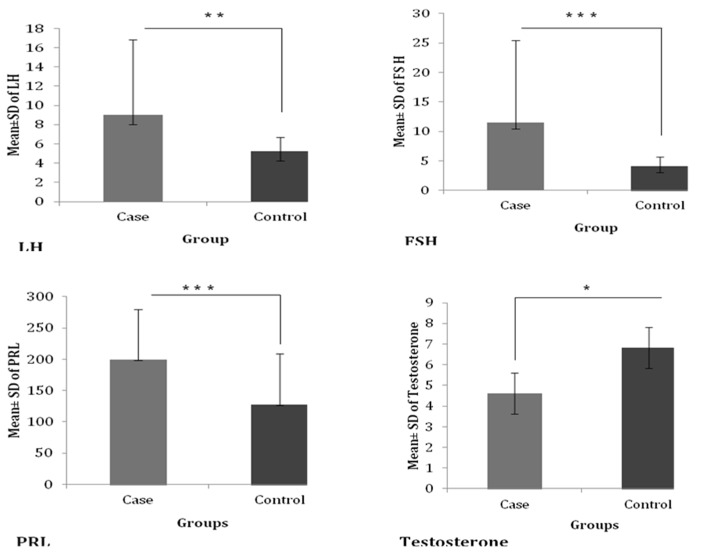
Serum hormone levels (mean ± SD) in case and control groups

In our study, most of the infertile cases (40%) were observed in the age group of 26-30 years followed by 31-35 years (27.3%), 36-40 years (20%) and 21-25 years (12.7%). Moreover, the majority of the controls were also related to 26-30 years (30.5%) and 31-35 years (29.5%) age groups. This difference was associated significantly (p=0.002) (Figure 1) The mean (±SD) semen profile (semen volume, sperm count, motility and morphology) of all infertile males is summarized in Table 1. The semen volume (ml) of azoospermic males was 2.40±1.03, whereas in oligozoospermia, asthenozoospermia, and normozoospermia it was analyzed as 2.63±1.17, 2.37±0.74 and 2.67±0.98, respectively. Moreover, in all the patients of oligozoospermia, asthenozoospermia and normozoospermia subgroups, sperm count (106/ml) were estimated as 5.11±5.44, 71.6±49.32 and 52.77±26.31, the percent motility was 56.32 ± 9.74, 16.99±10.63 and 62.71±8.55 and percent morphology was 60±13.86, 62.40±12.58 and 78 ±13.41, respectively. The mean (±SD) levels of different hormones in overall cases vs. controls were analyzed as LH (9.02±7.81 vs. 5.22±1.45 mIU/ml), FSH (11.45-14.02 vs 4.09±1.62 mIU/ml) and PRL (199.08±80.79 vs 127.23±81.64 ±IU/ml). These hormone levels were significantly higher in cases compared to controls. However, a significantly low level of Testosterone was observed in cases and found associated with male infertility (4.62±2.03 vs. 6.82±2.79 ng/ml) (Figure 2). In different subgroups of infertile males, mean (±SD) levels of LH, FSH, and PRL in azoospermia and oligozoospermia were 11.77±10.28, 16.82±17.27, 197.55±83.09 and 7.64±3.21, 9.95±13.94, 215.48±82.03, respectively. However, these hormones level in asthenozoospermic and normozoospermic males were 6.41±3.39, 5.96±4.80, 190.20±77.33 and 5.94±2.02, 5.14±2.50, 225.62±77.60, respectively as against 5.22±1.45, 4.09±1.62 and 127.23±81.64 in controls. So, LH, FSH, and PRL levels were found elevated significantly in azoospermic, oligozoospermic and asthenozoospermic infertile males while normozoospermic males had significantly higher levels of FSH and PRL in compared to fertile controls. Although, mean serum testosterone levels in azoospermia, oligozoospermia, asthenozoospermia, and normozoospermia were found as 4.49±1.97, 4.06±0.87, 4.70±1.97 and 6.21±3.45, respectively while in controls it was 6.82±2.79. Serum testosterone levels were significantly decreased in all the subgroups excluding normozoospermic subgroup (Table 2). In case group, Pearson's correlation test showed a significant negative correlation of LH and FSH levels with sperm count (r= -0.22; p=0.001 and r= -0.25; p<0.001), sperm motility (r= -0.21; p=0.002 and r= -0.22; p=0.001) and sperm morphology (r= -0.31; p<0.001 and r= -0.33; p<0.001), respectively. However, a poor negative correlation was also found between serum PRL and sperm motility (Table 3). In Receiver Operating characteristics (ROC) curve analysis, serum hormones; LH (AUC=0.638, Z=4.31, p<0.001), FSH (AUC=0.710, Z=7.19, p<0.001), Testosterone (AUC=0.718, Z=6.72, p<0.001), PRL (AUC=0.764, Z=9.95, p<0.001) showed the significant prediction for overall male infertility in comparing with controls. However, among serum hormones in different subtypes of infertility, only PRL showed the significant prediction of Normozoospermia (AUC=0.836, Z=4.916, p<0.001) at a cut off value of >119.04 µIU/ml with 92.86% sensitivity (95% CI=66.1-98.8) and 65.00% specificity (95% CI=54.8-74.3).

## Discussion:

Male factor is responsible for 40-50% of all infertility cases and affects about 7% of all men. It is mainly due to abnormalities in semen parameters and quality, which are used as a measure of normal spermatogenesis [12]. The spermatogenesis depends on the proper functioning of a complex action of reproductive hormones but alterations in the level of these hormones lead to abnormal spermatogenesis and cause infertility. Therefore, endocrinological evaluation is important in infertile males for proper diagnosis and treatment. In our study, the majority of the patients were in the age group of 26-30 years (40%). Another study by Bhale et al. (2013) also found most infertile patients in the age group of 25-40 years, which is in concordance to our findings [7]. As similar to a previous report by Najar et al (2010), the present investigation also confirmed that most of the infertile patients were with azoospermia (46.2%) followed by asthenozoospermia (34.5%), oligozoospermia (12.7%) and normozoospermia (6.4%) [6]. However, few similar studies of Nigeria and India as well reported the higher frequency of oligozoospermia while azoospermia was found in less percentage [12,13,14]. On the contrary, some other Indian studies found an equal number of oligozoospermic and azoospermic patients in their study cohorts [7,15]. The discrepancies in the prevalence of azoospermia may be due to the bilateral ductal obstruction or failure of spermatogenesis. Despite that frequency of asthenospermia was found 65% among infertile males in Jordan population which is approximately 2 times higher than the present study while Kumar et al. (2015) found 19.35% asthenozoospermic patients in their investigations [12,16]. Moreover, in this investigation, 6.4% of patients were diagnosed with normozoospermia, which is almost similar to the findings of Eniola et al. (2012) [14]. We quantified serum concentration of LH, FSH, Testosterone, and PRL in all the individuals; Elevated levels of LH, FSH and decreased levels of testosterone were significantly associated with azoospermic, oligozoospermic and asthenozoospermic patients in comparing with fertile controls. These outcomes indicate abnormal spermatogenesis of infertile males. Some previous studies are in partly support to our results. They reported elevated serum levels of LH, FSH and lower levels of testosterone [6,7]. However, no significant difference in mean serum levels of testosterone between infertile cases and fertile controls was found by some authors [14,15]. Pearson's correlation test showed no significant correlation between serum hormones and semen volume in all infertile patients. However, the LH and FSH hormones were inversely correlated with sperm count, motility and morphology. Interestingly, testosterone was positively correlated with all semen parameters. Since, FSH is known to have a direct role in immature testis development as well as in maintaining spermatogenesis, while LH is required to promote spermatogenesis indirectly via testosterone. Though, elevated LH and FSH levels stimulate Leydig and Sertoli cells for balanced production and secretion of testosterone thus enhancing spermatogenesis. At definite plasma threshold of gonadotropins, high LH and FSH levels generate a negative feedback effect on hypo thalamopituitary-gonadal axis. Therefore, the serum testosterone level becomes low or normal. Accordingly previous reports we also found that the semen parameters vary with the level of testosterone [7,17]. A higher concentration of FSH is a reliable indicator of germinal epithelial damage and found to associate with a decrease in the mean semen parameters. The increased level of FSH and decreased level of testosterone detected in azoospermia and oligozoospermia support the perception that inverse relationships exist between FSH elevation and spermatogenesis reduction [18]. Interestingly, the variations in serum levels of FSH and LH may be a possible cause of abnormal spermatogenesis. Our data determine the role of PRL on semen parameters and reported that its mean serum concentration in all the subgroups (azoospermic, oligozoospermic, asthenozoospermic and normozoospermic) was significantly elevated in comparison to control group which is in accordance of some previous investigations [9,16] However, Pearson's correlation analysis in the overall infertile patients depicted an inverse correlation between serum PRL level and sperm motility. Contradictorily, some references concluded that hyperprolactenemia was a rare cause of male infertility [10]. The increased level of PRL altered the feedback mechanism on the hypothalamus, further inhibits the pulsatility of GnRH secretion, thereby reducing the pulsatile release of LH, FSH and testosterone, which is a major cause of disrupted spermatogenesis, abnormal sperm motility and quality [16, [19,20]. Interestingly, ROC analysis depicted serum PRL as a predictor for male infertility. Ultimately, the reason for different findings of serum PRL level is still ambiguous. There is a need for more studies to confirm our findings.

## Conclusions:

Data shows the significant association of elevated serum levels of LH, FSH, PRL and low level of Testosterone with male infertility. Moreover, elevated serum levels of LH, FSH and PRL were significantly associated with azoospermia, oligozoospermia and asthenozoospermia while in normozoospermic infertile individuals serum FSH and PRL were elevated. Serum PRL is identified as a diagnostic marker for infertility in men from ROC data analysis. These findings are interesting for further confirmation using more patients of multiple cohorts.

## Figures and Tables

**Table 1:** Semen profile in different subtypes of infertile males.

Subtypes	n= 220 (%)	Semen Vol. (ml) (Mean ±SD)	Sperm count (10^6^/ml) (Mean ±SD)	% Motility (Mean±SD)	%Morphology (Mean ±SD)
Azoospermia	102 (46.4)	2.40 ±1.03	0	0	0
Oligozoo-spermia	28 (12.7)	2.63 ±1.17	5.11 ±5.44	56.32 ±9.74	60 ±13.86
Asthenozoo-spermia	76 (34.5)	2.37 ±0.74	71.6±49.32	16.99 ±10.63	62.40 ±12.58
Normozoo-spermia	14 (6.4)	2.67 ±0.98	52.77±26.31	62.71 ±8.55	78 ±13.41

**Table 2:** Serum hormone levels in all infertile subgroups and fertile controls

Groups	Hormones Level			
	LH (mIU/ml) Mean±SD (Range)	FSH (mIU/ml) Mean±SD (Range)	Testo (ng/ml) Mean±SD (Range)	PRL (µIU/ml) Mean±SD (Range)
Fertile controls; n= 220	5.22±1.45 (0.98-8.67)	4.09±1.62 (1.22-12)	6.82±2.79 (1.12-13.87)	127.23±81.64 (6.12-376.7)
Azoospermia; 102	11.77±10.28 * (0.64-43.03)	16.82±17.27* (1.07-56.52)	4.49±1.97* (0.22-9.11)	197.55±83.09* (44.48-381.6)
Oligozoospermia; 28	7.64±3.21*(3.61-13.82)	9.95±13.94*(2.11-57.29)	4.06±0.87*(2.11-6.08)	215.48±82.03*(67.15-383.72)
Asthenozoospermia; 76	6.41±3.39*(1.78-17.24)	5.96±4.80*(1.09-31.80)	4.70±1.97*(0.89-10.81)	190.20±77.33*(60.4-345.56)
Normozoospermia; 14	5.94±2.02(2.91-8.96)	5.14±2.50*(1.63-9.64)	6.21±3.45(3.18-15.04)	225.62±77.60*(93.7-324.15)
*Significant, p-value obtained by Unpaired t-test, n: number

**Table 3:** Correlation of LH, FSH, Testosterone, PRL hormones with semen profile of infertile patients

Semen profile	LH	FSH	Testosterone	PRL
Semen volume r p-value	-0.01 (0.87)	-0.001 (0.98)	0.04 (0.59)	0.01 (0.85)
Sperm count r p-value	-0.22 (0.001*)	-0.25 (<0.001*)	0.11 (0.098)	0.07 (0.304)
Sperm motility r p-value	-0.21 (0.002*)	-0.22 (0.001*)	0.09 (0.171)	-0.06(0.353)
Sperm morphology r p-value	-0.31 (<0.001*)	-0.33 (<0.001*)	0.04 (0.516)	0.05 (0.437)
*Significant, p-value obtained by Pearson correlation test (twotailed),r: correlation coefficient

**Figure 3:**
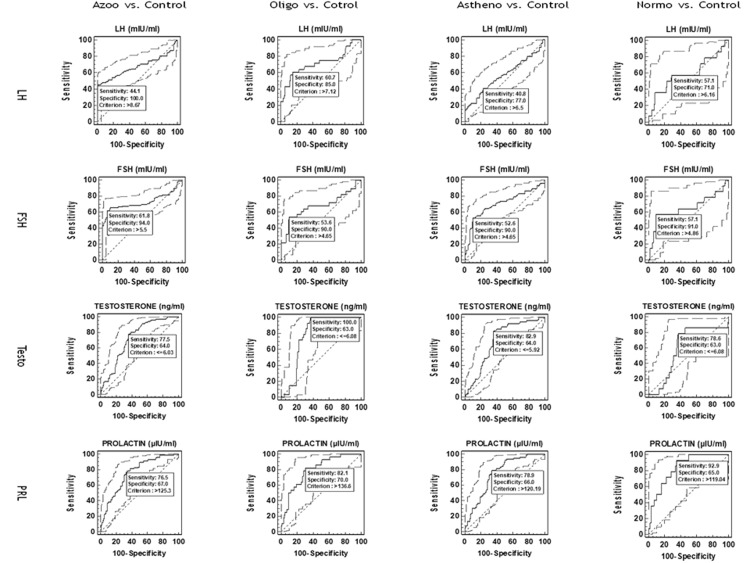
Diagnostic accuracy of different variables in predicting male infertility using ROC curve analysis
